# What Students Want to Hear After Failure

**DOI:** 10.3390/bs16071046

**Published:** 2026-06-23

**Authors:** Al Robiullah, Rebecca Gold, Kelsey Collins, Daeun Park, Gerardo Ramirez

**Affiliations:** 1 Department of Educational Psychology, Ball State University, Muncie, IN 47306, USArlgold@bsu.edu (R.G.);; 2 Department of Psychology, Sungkyunkwan University, Seoul 03063, Republic of Korea

**Keywords:** academic failure, feedback, emotional validation, student preferences, faculty responses, higher education

## Abstract

Academic setbacks are common in college, yet instructor responses to poor performance vary widely and may shape students’ motivation, emotional reactions, and perceptions of faculty support. Prior work suggests that supportive communication matters, but less is known about which types of messages students prefer after academic failure or whether faculty accurately anticipate these preferences. The present research examined how college students and instructors evaluate different instructor responses to a disappointing exam grade and assessed alignment between student preferences and faculty perceptions. Using a mixed-methods design, college instructors and undergraduate students responded to parallel vignette scenarios involving a poor exam outcome and rated brief instructor comments representing three response types: solution-focused, emotional validation, and interpersonal affirmation. Participants also provided open-ended responses describing what they would say to a student or want to hear from an instructor. Across two studies, students rated affirmation as most effective, validation as moderately helpful, and solution-focused responses as least effective, despite perceiving solution-focused comments as most common in actual classrooms. Faculty in our sample rated validation and affirmation as more effective than solution-focused responses but primarily generated strategy-focused advice in their own responses. Faculty correctly anticipated students’ preference for encouragement but rarely offered such messages. These findings point to a gap between what faculty believe students value and what they typically communicate following academic setbacks, suggesting that incorporating brief affirming and emotionally responsive messages may strengthen student–teacher relationships by signaling care, understanding, and support in moments of academic difficulty.

## 1. The Support Gap in Instructional Relationships: Examining Discrepancies Between Student Preferences and Faculty Perceptions of Academic Support

Moments of academic failure can strengthen or strain the relationship between professors and students (Akolgo et al., 2025). Because faculty serve as the primary source of feedback and the gatekeepers to future opportunities, their responses to student struggles significantly shape students’ emotional reactions, motivation, and sense of belonging in the field (Dweck, 2006; Yeager & Dweck, 2012). However, a significant support gap often exists in these interactions. While professors may approach struggling students through an instructional lens by focusing on error correction, students may seek emotional understanding and expressions of belief in their potential. Understanding which responses students value, and whether faculty accurately perceive these preferences, can be helpful for improving communication and persistence in college settings.

Historically, most models of emotion regulation have focused on intrapersonal processes, emphasizing how individuals modify their own thoughts through strategies like reappraisal (Gross, 2015; Bonanno & Burton, 2013). While these frameworks clarify how regulation unfolds within the individual, they offer less insight into how emotions are managed in social contexts where individuals rely on others to navigate distress. Recent work on interpersonal emotion regulation addresses this gap by treating supportive interactions as part of the regulation process itself, rather than as external influences (Zaki & Williams, 2013; Nozaki & Gross, 2025). From this perspective, a professor’s response may serve as a regulatory act that actively shapes how a student interprets and acts upon their distress.

This regulatory role requires faculty to navigate both instructional and interpersonal demands, a task that becomes particularly complex following academic setbacks. How faculty manage this tension can manifest in at least three distinct communicative pathways: prioritizing technical problem solving, providing emotional validation, or offering interpersonal affirmation. Examining these specific approaches reveals how different messages either meet or miss the regulatory needs of students in crisis.

### 1.1. The Problem-Solving Default

Professors, trained as disciplinary experts and socialized into roles as knowledge transmitters, may instinctively respond to academic failure as a technical problem requiring correction rather than as an emotional experience requiring support (Hargreaves, 1998; Zembylas, 2007). This default may be reinforced by institutional pressures to maintain standards and by beliefs that emotional support falls outside the scope of faculty responsibilities (Zembylas, 2007). Hence, faculty may default to strategy and error correction in ways that unintentionally bypass the emotional impact of failure (Gottman, 1994; Hargreaves, 1998; Zembylas, 2007).

These solution-focused responses may, from a regulatory standpoint, represent attempts to alter the situation by reducing distress indirectly through problem resolution rather than through emotional acknowledgment. While such responses can provide concrete guidance that students find useful (Voelkel et al., 2020), bypassing emotional acknowledgment entirely may leave some students feeling unseen.

We distinguish between dismissive invalidation, which explicitly minimizes emotional experience (e.g., “It’s not that bad”), and solution-focused responses, which direct attention toward understanding mistakes, correcting errors, or improving future performance (e.g., “Let’s review what you got wrong”). While dismissive invalidation is generally experienced as rejecting because it denies or minimizes a person’s emotional reaction (Lipnevich et al., 2025), solution-focused responses may be perceived more ambiguously (Williams, 2024). Such responses provide concrete guidance that students may find useful (Collier & Morgan, 2008), yet they do not explicitly acknowledge the emotional impact of academic setbacks. As a result, some students may experience these responses as helpful and supportive, whereas others may perceive them as overlooking an important emotional dimension of the experience. Whether students primarily value the practical guidance offered by solution-focused responses or prefer responses that first address their emotional reactions remains an open empirical question. To understand why solution-focused responses may sometimes fall short, we next consider how emotional experiences are regulated in social interactions.

### 1.2. Emotional Validation

If professors’ default problem-solving responses may inadvertently bypass students’ emotional needs, what might a more emotionally attuned response look like? Validation theory offers one framework. When students face academic setbacks, they experience not only threats to their competence but may also question whether their emotional reactions are legitimate or appropriate (Linehan, 1997). Validation refers to recognizing and legitimizing a person’s emotional response in a way that communicates understanding and acceptance. A validating professor might say, “I can see why this is frustrating; that reaction makes sense.” Rather than immediately offering solutions or minimizing the emotional experience, validation first acknowledges the person’s internal state as understandable given their circumstances.

Indeed, validation has been found to be a helpful form of emotional support in parenting, caregiving, and education. Across these settings, validation predicts stronger relationships and better emotional regulation (Campbell et al., 2024; Krause et al., 2003; Shenk & Fruzzetti, 2013; Linehan, 1993, 1997). Experimental work also shows that validation reduces physiological stress following difficult tasks (Shenk & Fruzzetti, 2011) and can lead to a variety of pro-social outcomes with adult caregivers (Jeon & Park, 2024; Kim et al., 2026).

Although these findings suggest that validation can improve emotional outcomes, little research has examined whether students actually prefer validation to other types of support after academic failure. Most studies test whether validation is effective, not whether it aligns with what students most want to hear. In the context of academic setbacks, validation specifically targets students’ emotional reactions to failure by communicating that those reactions are understandable and legitimate.

### 1.3. Affirmation

Beyond validation, which acknowledges students’ emotional experiences, professors might also communicate confidence in students’ abilities and potential. Affirmation refers to interpersonal messages that express belief in a person’s competence, capability, or worth, particularly in moments of doubt or failure. Both validation and affirmation express care, yet they differ in their function and what they focus on. Validation communicates that a student’s emotional response is understandable and legitimate (“your feelings make sense”), whereas affirmation communicates confidence in the student’s competence and future success (“you are capable”). Unlike validation, affirmation does not primarily seek to reduce emotional distress. Rather, it seeks to restore feelings of competence that may be threatened by failure.

Early research on affirmations examined how verbal encouragement could foster learning and resilience, whether through self-statements like “I am capable” or through teacher messages like “You can do this” (Coué, 1922; Paulhus, 1993). However, research on praise and mindset cautioned against messages that emphasize ability, linking them to fixed beliefs and maladaptive responses to failure (Mueller & Dweck, 1998; Dweck, 2006; Brummelman & Dweck, 2020). Recent scholarship suggests that the value of affirmation may have been dismissed too quickly. Wood and Harris (2013, 2016) argue that direct expressions of high expectations and intellectual capability (“You are smart,” “You are capable,” “Your mind is brilliant”) carry distinct social and psychological weight for students who rarely hear such affirmation. Across mixed-methods studies, Wood shows that adults who affirm students’ intellectual worth, rather than focusing solely on effort, provide a protective signal that counters deficit narratives.

Although conceptually distinct from affirmation, research on wise feedback highlights the value of communicating confidence in students’ abilities. Wise feedback typically combines critical feedback with an explicit statement of high standards and confidence in the student’s capacity to meet those standards (Yeager et al., 2014). Unlike affirmation, which can occur in the absence of evaluation or criticism, wise feedback is specifically designed to shape how students interpret corrective feedback. Studies show that pairing criticism with a brief statement of confidence increases trust, motivation, and receptivity to feedback (Yeager et al., 2014). In part, these effects may stem from the message’s affirming component, which communicates that the student is capable, valued, and expected to succeed (Basileo et al., 2024; Wang et al., 2025). These findings suggest that when a professor says “I believe you can do this,” the message functions not only as encouragement but also as social confirmation of competence from a credible source.

Thus, when students experience academic failure, professors face a choice among various qualitatively different response pathways: they can address the technical problem (solution-focused feedback), acknowledge the emotional experience (validation), and/or restore threatened competence (affirmation). Importantly, these response types are not mutually exclusive. Rather, the present work focuses on professors’ initial responses to student distress, which may shape students’ willingness to engage further (e.g., seeking help, attending office hours), where more detailed problem solving can occur.

### 1.4. The Current Study

The literature reviewed above reveals three theoretically distinct response pathways available to professors following academic failure. Solution-focused responses primarily address the instructional problem by directing attention toward strategies, corrections, and future performance. Validation addresses the emotional dimension of the experience by acknowledging that students’ reactions are understandable and legitimate. Affirmation addresses threatened competence beliefs by communicating confidence in students’ abilities and potential. Although these approaches may overlap in practice and may be combined within a single interaction, they differ in the psychological needs they are designed to address. Wise feedback represents a related but distinct approach in which critical feedback is paired with an explicit statement of high standards and confidence in the student’s ability to improve (Yeager et al., 2014). The present study focuses specifically on students’ and instructors’ perceptions of solution-focused responses, validation, and affirmation following academic setbacks.

Study 1 provided an initial exploratory examination of how college instructors conceptualize and respond to student academic distress. Forty-five instructors rated the effectiveness and frequency of different response types and described both what they would say to a struggling student and what they believed students most wanted to hear. Although the instructor sample was modest, obtaining faculty participation in communication-focused survey research is often substantially more difficult than recruiting student samples, particularly when participation requires open-ended responding. Importantly, the faculty data served a comparative purpose to identify whether faculty perceptions and reported practices aligned with student preferences. Study 2 paralleled this design with 153 college students who evaluated and described their preferred responses following a disappointing grade. By collecting parallel data from both groups using the same materials, the studies identify areas of convergence and divergence in perceptions of academic support.

Importantly, the present work does not assume that what students prefer is always identical to what produces optimal learning outcomes. Preferences and effectiveness can diverge, as illustrated by well-known cases such as students’ endorsement of learning styles or cramming despite limited evidence for their benefits (Pashler et al., 2008; Dunlosky et al., 2013). However, this divergence does not render preferences irrelevant. Within social emotion regulation, individuals consistently prefer validating responses because they are perceived as more comforting, even when alternative responses may be equally effective at shifting emotional states (Sahi et al., 2023). More critically, the relational context in which feedback is delivered shapes whether that feedback is received at all. Responses that signal understanding and responsiveness reduce social threat, build trust, and increase openness to guidance (Elliott et al., 2018; Reis, 2012). Experimental evidence reinforces this, such as when critical feedback is paired with an explicit signal of confidence in the student’s capacity to improve; students are substantially more likely to engage with and revise based on that feedback, even when the feedback content is otherwise identical (Yeager et al., 2014). Guidance delivered without acknowledgment of the student’s experience, by contrast, can feel dismissive and limit receptivity to information that would otherwise be useful (Steindl et al., 2017). Students’ preference for affirmation or validation may therefore reflect sensitivity to the relational conditions that determine whether instructional feedback becomes usable.

The present study addressed the following research questions:

RQ1: How do faculty and students evaluate the effectiveness of solution-focused feedback, validation, and interpersonal affirmation after academic setbacks?
RQ2: How do faculty and students perceive the frequency of these different response types in current practice?
RQ3: Where do perceptions align and misalign between (a) what faculty report doing, (b) what faculty believe students want, and (c) what students actually prefer?

## 2. Study 1 Methods

### 2.1. Participants

The instructor sample was intended to provide an initial exploratory comparison point for examining faculty perceptions and reported practices regarding student support following academic setbacks. Recruitment continued throughout the summer and fall of 2025 in an effort to enroll the maximum feasible number of eligible instructors.

The sample constituted a convenience sample, which may introduce self-selection bias, as instructors with greater interest in teaching practices or student support may have been more likely to participate. To reduce this risk, recruitment messages were distributed broadly across multiple listservs and institutions rather than targeting specific subgroups. Participation was anonymous and voluntary, which was intended to reduce social desirability pressures and encourage honest reporting of instructional practices.

A total of 45 professors completed the online survey, a sample size comparable to or larger than many faculty-focused survey studies that require open-ended responding. Most participants taught at four-year universities (*n* = 30, 66.7%), with the remaining participants teaching at two-year community colleges (*n* = 15, 33.3%). Teaching experience ranged from 1 to 35 years (M = 12.6, SD = 8.4), reflecting a mix of early-career and senior faculty across higher education contexts. In terms of gender identity, 26 participants (57.8%) identified as female, 17 (37.8%) as male, and 2 (4.4%) selected another gender or a list of combined genders.

### 2.2. Design

This mixed-methods study examined faculty and student perspectives on three types of instructor responses to academic setbacks: solution-focused responses (problem solving without emotional acknowledgment), validation (acknowledging emotions), and interpersonal affirmation (expressing belief in capability).

### 2.3. Procedure

The study was administered online through Qualtrics and approved by the Institutional Review Board (IRB-1842888-1). Participation was voluntary, anonymous, and limited to current college instructors. After providing informed consent, participants completed the survey at their convenience.

The survey began with a short vignette in which professors were asked to imagine handing back exam scores and noticing a student who looks visibly disappointed with their own grade. Participants were then instructed to picture how they might respond in that moment and to answer two open-ended questions. The first asked what they would say to the student, and the second asked what they believed the student would most want to hear. These prompts elicited qualitative data about professors’ spontaneous communication styles and their assumptions about students’ emotional and motivational needs after academic setbacks.

After completing the open-ended section, participants proceeded to a rating task. They were shown a randomized list of brief instructor comments that reflected things other professors might say to a disappointed student in the same situation. These statements had been pre-classified by the research team as examples of solution-focused responses, validation, or affirmation, although participants were not told these categories. For each statement, professors indicated whether they believed it was effective (helpful to the student) and how frequently such comments are used by professors in general. The randomized order of the statements ensured that ratings were not influenced by statement order.

We constructed statements representing each response type based on theoretical definitions. Interpersonal affirmation statements expressed belief in the student’s capability and worth, designed to restore threatened competence by communicating that an expert recognizes the student’s potential (Yeager et al., 2014; Essien & Wood, 2021). Examples included: “Your hard work and dedication to your studies is admirable and will pay off in the long run” and “You are capable of achieving your goals.”

Validation statements acknowledged and normalized the student’s emotional experience. Based on Linehan’s (1997) validation theory, these statements communicated that feelings were understandable and legitimate (Shenk & Fruzzetti, 2011). Examples included: “Your feelings of disappointment are completely understandable given how much effort you put into preparing for the exam” and “It’s not uncommon for students to feel discouraged after a poor exam result.”

Solution-focused statements provided corrective feedback without acknowledging emotions or affirming capability. These statements bypassed emotional acknowledgment by immediately pivoting to problem solving. Examples included: “Tell me about how you studied for the exam and I can give you some advice for next time” and “Take this exam result as feedback and use it to improve how you study for exams”.

The entire procedure took approximately 15–20 min to complete. All responses were recorded through Qualtrics and later downloaded for quantitative and qualitative analysis.

### 2.4. Transparency and Openness

For Study 1, we recruited the maximum feasible sample of eligible college instructors during the recruitment period. Faculty recruitment is often more difficult than student recruitment, particularly in studies requiring both quantitative ratings and open-ended written responses. Accordingly, Study 1 was primarily intended as an exploratory comparison study designed to identify broad patterns of convergence and divergence between faculty perceptions and student preferences rather than to produce precise population estimates.

For Study 2, we recruited the maximum feasible sample of students within the available data-collection budget. We did not conduct an a priori power analysis for either study. As a conservative sensitivity analysis, we treated the focal comparisons as independent two-group proportion tests with alpha set at 0.05. Under these assumptions, Study 2 (*N* = 153) had 80% power to detect effects of approximately Cohen’s h = 0.32, corresponding to a difference of roughly 15 to 16 percentage points when baseline endorsement was near 50%. Study 1 (*N* = 45) had 80% power to detect effects of approximately h = 0.59, corresponding to a difference of roughly 25 to 30 percentage points under similar conditions. Because the primary analyses used mixed-effects models that leveraged repeated ratings across multiple statements per participant, these sensitivity estimates are conservative and likely underestimate the available information in the dataset.

All data and materials for both studies are publicly available (Open Science Framework, 2026). No data were excluded beyond removing incomplete responses that did not meet basic attention-check or consent requirements, which are documented in the posted dataset. All materials, including survey instruments and response statements, are also available at the same repository link. Data were analyzed in R (R Core Team 2015) using the packages lme4 (Bates et al., 2015) and tidyverse (Wickham et al., 2019). The research design, hypotheses, and analytic plan were developed prior to data analysis, but no aspect of the research was formally preregistered in an external registry.

## 3. Study 1 Results

### 3.1. Qualitative Analysis

We conducted a thematic analysis to better understand participants’ written responses and to complement the quantitative findings. The analysis followed Braun and Clarke’s (2006) six-phase framework for thematic analysis. The first and second authors independently reviewed all open-ended responses to identify recurring ideas and patterns in how professors described their own reactions to struggling students and how they believed students would prefer to be supported. Both authors read the responses multiple times to become familiar with the data and note initial impressions. The first author developed an initial coding framework, which was refined collaboratively throughout the analytic process. The framework was informed by both inductive patterns emerging from the data and deductive categories derived from the quantitative analyses.

To ensure transparency, each open-ended response was assigned a primary code based on its dominant theme, with a secondary code applied when a response contained meaningful elements of more than one category. Four categories emerged. Solution-focused responses emphasized academic strategies, error correction, or performance improvement without first addressing the student’s emotional experience (e.g., faculty: “Tell me about how you studied. What material did you focus on?”; student: “Advice on where I went wrong and tips on where I could improve”). Validation responses directly acknowledged emotions and communicated empathy or understanding (e.g., faculty: “It is understandable that the student is disappointed and they should be allowed to process that as they wish”; student: “I can tell you are unhappy with the grade you received and I’m sorry”). Affirmation responses expressed confidence in the student’s ability, effort, or potential (e.g., faculty: “Your value as a student isn’t determined by your grades” and “I am confident they could improve and succeed”; student: “You are capable of achieving your goals. Don’t give up”). Finally, Neutral/Other responses included procedural, policy-driven, or explicit non-responses that did not fit the preceding categories (e.g., faculty: “I won’t say anything”; student: “Nothing that was not explained above”). No responses were classified as dismissive invalidation.

After establishing the coding framework, the two authors independently coded the full dataset. Agreement between coders was high (93.3%). Discrepancies were resolved through discussion and consensus. The resulting thematic summary was then reviewed by a third author, who confirmed that the themes accurately reflected the content of the responses. This iterative process strengthened the reliability and credibility of the analysis.

### 3.2. Qualitative Findings

**Figure 1:**
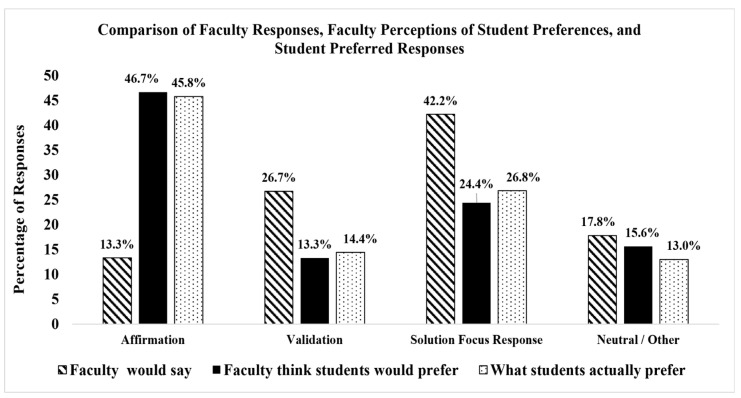
Comparison of themes identified in faculty-generated responses, faculty predictions of student preferences, and student preferred responses following a disappointing exam grade. Values represent the percentage of open-ended responses coded into each thematic category.

Open-ended responses to the question “If you were the professor in the scenario, what would you say?” revealed four main themes: Solution-focused, Validation, Affirmation, and Neutral/Other. These categories capture the range of ways professors described responding to struggling students. The frequency of each response type differed significantly, χ^2^(3, *N* = 45) = 8.78, *p* = 0.032. Specifically, Solution-focused responses were the most common theme among professors (see Figure 1).

Most professors (42.2%, *n* = 19) described responses centered on problem solving, such as offering strategies or reviewing study habits: “Tell me about how you studied. What material did you find most difficult?” and “Let’s talk about strategies for preparing better next time.” Although these comments were well-intentioned and academically helpful, they were coded as solution-focused responses because they moved quickly toward addressing the problem without first recognizing the student’s emotions.

About a quarter of professors (26.7%, *n* = 12) offered emotionally attuned responses that acknowledged students’ feelings and normalized their reactions. For instance, “I understand that you are disappointed by this score” and “I know you worked really hard.” These responses were coded as validation because they directly acknowledged the student’s emotional experience.

A smaller portion (13.3%, *n* = 6) provided affirming messages that reassured students of their value and potential, such as “Your value as a student isn’t determined by your grades” and “I am confident you will succeed.” Finally, some professors (17.8%, *n* = 8) reported that they would remain silent or limit their response due to policy concerns, offering statements such as “Nothing” and “I would not want to risk violating FERPA regulations.”

Within the present sample, professors more frequently prioritized offering solutions over directly addressing emotions when speaking to struggling students. While this reflects a desire to be helpful, it often skips the validating or affirming step that may be necessary before students are ready to engage with feedback or improvement strategies.

Interestingly, however, when asked “What do you think students would most want to hear after receiving a disappointing grade?”, professors showed a notably different pattern. The chi-square test again indicated a highly unequal distribution across categories, χ^2^(4, *N* = 45) = 25.56, *p* < 0.001 (see Figure 1). Affirmation (47%) emerged as the most common type of message professors believed students preferred. Typical examples included statements such as “Don’t be discouraged, you’re capable” and “You worked hard on this, and there are still opportunities to improve.”

About one quarter (24.4%, *n* = 11) imagined that students would want direct guidance on how to improve. Examples included “Let’s review your answers” and “Here’s how you can improve.” While constructive, these statements were coded as solution-focused responses for the same reason as before: they centered on problem solving rather than emotional acknowledgment, assuming students primarily sought correction rather than understanding. Only a smaller group (13.3%, *n* = 6) expected that students would appreciate emotional recognition, offering examples like “I noticed you’re upset, it’s okay to feel disappointed.” These were coded as validation because they acknowledged and normalized students’ feelings. The remaining professors (15.6%, *n* = 7) gave context-dependent or neutral responses such as “It depends on the student.”

### 3.3. Quantitative Findings

We analyzed the survey data using mixed-effects logistic regression models implemented in the lme4 package in R (Bates et al., 2015). This approach was chosen because each professor rated multiple statements, and each statement was rated by multiple professors. These repeated and overlapping ratings created a crossed data structure, meaning that ratings were related both to the individual professor who made them and to the statement being rated. Mixed-effects models appropriately account for this dependency by including random effects for both professors and statements, preventing biased estimates that can occur when treating all ratings as independent.

Before running the main analysis all major statistical assumptions were evaluated and met. Specifically, variance inflation factors (VIFs) indicated no multicollinearity (<1.01), random effects showed nonzero variance, the models converged without singularity issues, residual and influential diagnostics indicated no violations, and outcome distributions were balanced. We ran two separate models, one predicting whether each statement was rated as effective and another predicting whether it was rated as common. Each model was estimated using the following general specification:

model_X <- glmer(X ~ StatementType + (1|ResponseID) + (1|StatementID), data = dataset family = binomial)

In this model, X represents one of two binary outcomes: (a) whether a statement was rated as Effective (1 = yes, 0 = no), or (b) whether it was rated as Common (1 = yes, 0 = no). Fixed effects included Statement Type, which categorized each professor’s response as Validation, Affirmation, or Solution-Focused Responses. Validation served as the reference category, allowing direct comparisons of how the odds of endorsement for affirmation and solution-focused responses differed relative to validation.

Random intercepts were included for both ResponseID (professor) and StatementID (statement). The random intercept for professors accounted for differences in rating tendencies—some professors may consistently rate statements more positively or negatively than others. The random intercept for statements accounted for variability across items, such as certain statements being generally viewed as more effective or more common. For each model, we calculated the estimated coefficients (β), standard errors, and *p*-values. We then converted these estimates into odds ratios and 95% confidence intervals by exponentiating the coefficients. To make the results more intuitive, we also calculated predicted probabilities for each statement type using the inverse-logit transformation. These analyses allowed us to compare how professors perceived validation, affirmation, and solution-focused statements in terms of both effectiveness and commonness, while appropriately accounting for individual and item-level differences.

Faculty rated validation statements as significantly more effective than solution-focused statements (β = −1.56, SE = 0.43, *p* < 0.001, OR = 0.21). See Table 1. Validating statements had about a 45% probability of being rated as effective, compared to just 15% for solution-focused statements. This suggests that professors recognize that acknowledging students’ feelings is generally helpful, at least when presented with other professors’ responses. Affirmation, which focuses on encouragement and confidence in students’ potential, was also rated as effective (50% probability) and did not differ significantly from validation (β = 0.19, SE = 0.42, *p* = 0.65, OR = 1.21). In other words, professors saw both affirmation and validation as comparably supportive. Affirmation, however, was rated as much more effective than solution-focused responses (β = 1.75, SE = 0.43, *p* < 0.001, OR = 5.75), showing that professors view emotionally supportive responses as far more beneficial than corrective or critical ones.

When asked about how common each response type is in practice, professors reported the opposite pattern. Solution-focused statements were seen as the most frequent (β = 0.95, SE = 0.42, *p* = 0.023, OR = 2.58), with a 52% probability of being rated as common, compared to 30% for validation. This pattern suggests that professors in our sample perceived solution-focused responses as relatively common in higher education despite rating them as less effective than validation or affirmation. Affirmation, despite being viewed as helpful, was seen as rare in practice. It was rated as less common than both validation (β = −1.30, SE = 0.43, *p* = 0.003, OR = 0.27) and solution-focused responses (β = −2.25, SE = 0.43, *p* < 0.001, OR = 0.11), with only a 10% probability of being considered typical.

### 3.4. Study 1 Discussion

Study 1 showed a clear pattern in how professors think about supporting students after failure. When evaluating responses written by other instructors, professors viewed validation and affirmation as more helpful than comments that moved straight into advice or error correction. However, when asked to write what they would say in the moment, most offered solution-focused comments that did not acknowledge students’ emotions or confidence concerns. Only a small number used validating or affirming language.

This gap suggests that faculty in the present sample recognized the value of supportive messages but still tended to rely on problem-focused responses. These responses were typically oriented toward problem solving and often did not explicitly address students’ emotional experiences.

## 4. Study 2—The Student Perspective

Study 2 shifted the focus from professors to students to examine how learners interpret and evaluate different types of instructor responses after an academic setback. While Study 1 showed that professors often rely on solution-focused statements when responding to disappointed students, it remained unclear whether students experience these responses as helpful or discouraging. The fact that professors rated such statements as ineffective yet reported using them frequently suggests a disconnect between faculty assumptions and their behaviors. However, it is possible that students actually appreciate the practical guidance embedded in solution-focused comments, even when these lack emotional acknowledgment.

In this study, college students read the same vignette describing a poor exam performance and rated a series of instructor comments representing solution-focused responses, validation, and affirmation. As in Study 1, students rated each comment on two dimensions: how effective it would be in supporting them and how common such feedback is in real classrooms. They also described, in their own words, what they would most want to hear from a professor in that situation. This design enabled direct comparison between student preferences and faculty practices, highlighting where perceptions align and where communication gaps persist. More importantly, it allowed us to test whether solution-focused responses are experienced as helpful forms of guidance or as subtle forms of invalidation that undermine well-being and engagement.

## 5. Study 2 Methods

### 5.1. Participants

A total of 159 students began the survey through Prolific, an online crowdsourcing platform for collecting data. Six participants were excluded because they did not pass the attention check and did not continue the survey, resulting in a final sample of 153 students. Data were collected during the summer of 2025 through prolific. The sample size was determined by available funding for participant compensation and by recruiting the maximum number of eligible participants within the data collection period. Eligibility was based on self-identification as a college student enrolled in a two- or four-year institution of higher education. This recruitment approach introduces several potential sources of bias. First, reliance on Prolific may limit representativeness, as participants on online platforms tend to differ from the broader college population in ways such as age, motivation, and familiarity with research participation. Second, the use of self-reported eligibility may introduce classification error, as enrollment status was not independently verified. Third, participation was voluntary and compensated, which may have attracted students who are more attentive to surveys or more interested in academic topics, potentially introducing self-selection bias.

The composition of the sample further reflects potential limitations in generalizability. Although gender was relatively balanced (49.7% male, 48.4% female), the sample was predominantly White (68.6%), with smaller representation from other racial and ethnic groups. This distribution may limit the extent to which findings generalize to more diverse student populations. Additionally, the sample included a relatively large proportion of graduate students (41.2%), which may influence responses, as graduate students may differ from undergraduates in their experiences with academic feedback and their expectations of instructor support. The inclusion of participants who were no longer currently enrolled (5.2%) may also introduce recall bias, as their responses reflect retrospective interpretations rather than immediate reactions.

To partially address these concerns, the study recruited participants across a range of class standings, first-generation statuses, and institution types, with the goal of capturing variability in student experiences.

### 5.2. Design

Study 2 used a cross-sectional, mixed-methods survey design to examine how college students evaluate three types of instructor responses to academic setbacks: solution-focused responses (problem solving without emotional acknowledgment), validation (acknowledging emotions), and interpersonal affirmation (expressing belief in capability). Participants responded to a vignette describing a disappointing exam outcome, rated instructor comments, and provided open-ended responses about their preferred feedback.

### 5.3. Procedure

The student survey was administered online through Qualtrics and approved by the Institutional Review Board (IRB-1842888-1). Participation was voluntary, anonymous, and open to undergraduate and graduate students enrolled in U.S. colleges and universities. After providing informed consent, students completed the survey at their own pace, which took approximately 20 min to complete.

The survey began with a brief scenario describing an academic setback. Students were asked to imagine a virtually similar scenario to what was given to professors in study 1. They were asked to imagine sitting in a college classroom, receiving a lower-than-expected exam grade after studying hard and feeling disappointed. The vignette described the professor walking around, noticing the student’s discouraged expression, and then responding.

After reading this scenario, students viewed the same randomized list of hypothetical responses that were used in Study 1. The hypothetical professor responses were designed to represent three distinct types of responses: Solution-focused responses, Validation, and Affirmation. The labels were not shown to participants. For each statement, students selected whether they thought it was effective (would have a positive impact on a student) and/or frequent (something professors commonly say). 

After rating all statements, students were asked an open-ended question inviting them to describe what they would have preferred to hear from the professor in the initial situation. This item provided qualitative insight into how students themselves define supportive communication following an academic setback.

## 6. Study 2 Results

### 6.1. Quantitative Findings

#### 6.1.1. Effectiveness Ratings

Students rated affirmation statements as the most effective type of instructor feedback (see Table 2). Affirming comments were significantly more likely to be rated as effective than both validation and solution-focused responses. Affirmation had a 64% predicted probability of being rated as effective, compared to 43% for validation and 36% for solution-focused responses (β = 0.86, SE = 0.27, *p* = 0.001, OR = 2.36 vs. validation; β = 1.17, SE = 0.27, *p* < 0.001, OR = 3.22 vs. solution-focused responses).

Students viewed validation responses as moderately helpful but not clearly distinct from solution-focused responses in their overall ratings (β = −0.31, SE = 0.27, *p* = 0.25, OR = 0.73). This suggests that while students appreciate when professors acknowledge their emotions, they prioritize affirmation responses. In short, students find affirming responses most motivating, validating responses somewhat helpful, and solution-focused responses least effective.

#### 6.1.2. Frequency Ratings

When asked how often these types of responses occur in real classrooms, students reported a very different pattern (see Table 2). Solution-focused responses were rated as the most common response from professors, with a predicted probability of 64%, compared to 46% for validation (β = 0.74, SE = 0.20, *p* < 0.001, OR = 2.10). In contrast, affirmation was perceived as relatively rare, with only a 28% predicted probability of occurrence (β = −0.80, SE = 0.21, *p* < 0.001, OR = 0.45 vs. validation; β = −1.55, SE = 0.21, *p* < 0.001, OR = 0.21 vs. solution-focused responses).

### 6.2. Qualitative Findings

Students described in their own words what they would have preferred to hear from their professor after receiving a disappointing exam score. Their responses revealed four main themes: Affirmation, Validation, Solution-focused responses, and Neutral or Other. These themes provided insight into how students interpret supportive versus unsupportive feedback and helped contextualize the quantitative results.

The significant chi-square analysis result, χ^2^(3, *N* = 146) = 41.94, *p* < 0.001, confirms that students do not value all types of post-exam feedback equally but instead show clear preferences for certain forms of communication. Nearly half of all students (45.8%, *n* = 67) favored affirming messages (see Figure 1). One student wrote, “I would have wanted my professor to tell me that I can still do well in the class and that one test doesn’t define me.” Another added, “It helps when a professor reminds you that they believe in you and that you can still improve.” Students clearly valued messages that rebuilt their confidence and helped them stay motivated after setbacks. The next most common theme was solution-focused responses (26.8%, *n* = 39), which captured students’ desire for constructive academic guidance. For example, one student wrote, “Help me understand what I missed so I can do better,” while another said, “Show me how to study next time because what I did didn’t work.” These statements suggest that students appreciate when professors provide practical feedback.

A smaller portion of responses reflected validation (14.4%, *n* = 21). These students wanted professors to simply acknowledge and legitimize their feelings. One commented, “I would like them to acknowledge I am upset,” and another wrote, “It helps to know the professor understands what I am going through.” Such responses indicate that emotional acknowledgment, even without explicit encouragement or advice, can help students feel less isolated after a poor performance.

Finally, neutral or other responses (13%, *n* = 19) included brief or procedural remarks that did not express clear emotional or motivational content. Some students wrote that professors should “just tell me what to do next” or responded with “nothing.” These students seemed less interested in emotional support and more focused on efficiency.

## 7. Study 2 Discussion

Study 2 showed that students have clearer preferences than professors may assume. Students rated affirmation as the most helpful type of response after a poor exam. Many also described wanting this kind of support in their own words, often saying they needed some reassurance that their potential remained intact. Validation was seen as moderately helpful, while strategy-only comments were rated lowest, even though some students still appreciated practical guidance. These findings provide the necessary context for interpreting the faculty patterns from Study 1.

## 8. General Discussion

Across both studies, students consistently preferred affirming responses following academic setbacks, whereas faculty primarily generated solution-focused responses despite accurately anticipating students’ preferences. Nearly half of faculty believed students would want encouragement and expressions of confidence in their abilities, yet few spontaneously offered such messages when describing how they would respond to a struggling student. This pattern reveals a disconnect between what instructors believe students want to hear and the responses they naturally provide.

The reasons for this discrepancy remain unclear. One possibility is that students and instructors approach academic failure with different immediate priorities, with students seeking reassurance that their competence and potential remain intact and instructors focusing on performance improvement. Another possibility is that broader professional norms shape how instructors respond to student difficulties. Faculty are often trained as disciplinary experts and may be socialized to emphasize accuracy, improvement, and standards (Hargreaves, 1998; Zembylas, 2007). From this perspective, instructors may naturally gravitate toward corrective guidance even when they recognize the value of encouragement and affirmation. However, because these mechanisms were not directly measured in the present study, they should be viewed as tentative explanations rather than established conclusions.

Students consistently rated affirmation as the most effective response type. In their open-ended responses, nearly half of students spontaneously requested affirming messages, writing that they wanted professors to express belief in their ability to succeed or reassure them that one setback did not define their potential. Although less common than requests for affirmation, some students also expressed interest in practical guidance and emotional validation. Those who requested guidance wanted help understanding their mistakes or improving future performance, whereas those who requested validation wanted professors to acknowledge their disappointment or recognize that their feelings made sense.

Students showed interest in guidance but placed different weights on the timing and tone of that guidance. Many requested help understanding mistakes or adjusting study strategies in their open-ended responses. Even so, strategy-focused comments that bypassed emotional acknowledgment received the lowest effectiveness ratings. The present findings suggest that many students prefer reassurance about their potential before receiving advice on how to improve. When guidance arrives without this reassurance, it may feel hurried or out of sync with the emotional impact of the setback. The issue may not be whether professors offer strategies but when and how they do so.

Why might affirmation messages carry particular weight? When a professor expresses confidence in a student’s ability, the message comes from someone with recognized expertise in the domain. This gives interpersonal affirmation a particular authority. It signals that an informed observer sees potential beyond the setback and has the potential to enhance self-efficacy (Bandura, 1977; Zakariya, 2022). Studies on teacher feedback and academic belief formation show that messages that affirm strengths or express confidence can shape students’ sense of ability and influence their engagement (Cohen et al., 1999; Yeager et al., 2014; Wang et al., 2025; Basileo et al., 2024). Indeed, caregivers who use affirming statements elicit more cooperation (Campbell et al., 2024).

Affirmation may bridge emotional understanding and instructional effectiveness without requiring professors to adopt therapeutic language or engage in extended emotional discussion. It restores confidence efficiently and positions students to engage with corrective feedback once they feel capable of improvement. This may explain why affirmation outperformed both validation alone and solution-focused guidance alone. It addresses both the emotional threat and the competence threat that academic failure creates.

### 8.1. Limitations

Several limitations should be considered when interpreting these findings. An important consideration is that all findings were derived from responses to hypothetical vignette scenarios rather than observations of actual classroom interactions. Participants evaluated brief written exchanges in a standardized context, allowing direct comparison across response types but necessarily reducing the complexity of real instructor-student relationships. In actual classrooms, responses occur within ongoing relationships, vary in timing and delivery, and are influenced by contextual factors that cannot be fully captured in a vignette. Consequently, the present findings are best interpreted as evidence regarding participants’ perceptions and preferences rather than definitive evidence about how instructors and students behave during real academic setbacks.

This study also measured preferences and perceptions rather than actual outcomes. We do not know whether receiving affirmation improves persistence, performance, or well-being for students in our sample. Students may prefer affirming messages, yet they may benefit equally or more from other forms of support. Additionally, the scenarios were hypothetical and brief, lacking the emotional intensity and relational context of real classroom interactions.

Another limitation concerns the faculty sample in Study 1. Although the instructor sample size was modest, the study was intended as an exploratory comparison of faculty perceptions and reported practices rather than as a precise population estimate of higher education instructors broadly. The analyses also leveraged repeated ratings across multiple statements per participant using mixed-effects models, increasing the amount of information available within the dataset beyond a simple between-subjects design. Nevertheless, replication with larger and more diverse faculty samples would strengthen confidence in the observed patterns.

The vignette also did not clearly specify whether the interaction occurred in a public or private setting. This ambiguity is important, as instructors may respond differently depending on context, particularly when considering how to address students’ emotions. As a result, uncertainty about the setting may have influenced participants’ reported responses and contributed to variation in the findings.

Additionally, students completed the open-ended response after rating predefined statements. Because they were asked what they would have preferred to hear, their responses may have been influenced by the response options they had just seen. This makes it difficult to determine the extent to which these responses reflected fully independent views.

More importantly, our design cannot disentangle whether students disliked solution-focused responses because of their content or because of how it was delivered. The solution-focused statements explicitly bypassed emotional acknowledgment. Students who requested guidance in their open-ended responses may have assumed such guidance would be delivered with care and respect. Thus, we cannot determine whether the issue is strategic advice itself or providing it too quickly before students feel emotionally ready to receive it. Future research should examine whether sequencing matters by comparing affirmation followed by guidance against guidance alone.

Another limitation concerns the measurement approach. Participants made dichotomous judgments regarding the effectiveness and frequency of response statements. Although this approach reduced participant burden and facilitated comparison across a large number of statements, it provided less nuance than multi-point rating scales. Future research could employ more fine-grained measures of effectiveness, warmth, appropriateness, authenticity, and motivational impact.

Finally, contextual factors were not examined. Responses may differ depending on institutional culture, educational level, and setting. For example, faculty at small liberal arts colleges may be more encouraged to provide validation or affirmation, whereas instructors at large research-focused universities may rely more heavily on problem-solving feedback. Similarly, undergraduate and graduate students may differ in their expectations regarding instructor support and feedback following academic setbacks. Future studies using field observations and naturalistic classroom interactions would provide stronger evidence of ecological validity and could examine how different response types influence persistence, engagement, trust, and self-efficacy in real educational settings.

### 8.2. Implications for Practice

These findings have direct implications for faculty development and teaching practice. Departments can help instructors integrate affirmation into their feedback routines through simple adjustments to communication patterns. Rather than immediately pivoting to problem solving after noticing student disappointment, professors can begin by acknowledging effort and expressing belief in capability before offering guidance. For example, instead of opening with, “How did you study for this exam?” an instructor might say, “I know you are capable of improving. Let’s look at your preparation and identify strategies for next time.” This approach takes little additional time but may substantially change how students receive corrective feedback.

Faculty development workshops can use role-playing and peer observation to help instructors practice combining affirmation with technical guidance. Departments can model communication styles that pair rigor with encouragement, reinforcing that high academic standards and emotional support are not incompatible goals. These interventions need not require extensive training or shifts in teaching philosophy. They ask instructors to attend to the emotional and motivational dimensions of academic setbacks while continuing to provide the expertise-based guidance that defines their professional role.

## 9. Conclusions

Students facing academic failure want professors to affirm their capability and signal that poor performance does not define potential. The communication gap we identified reflects broader tensions in academic culture. Faculty are trained to diagnose errors but may neglect the emotional and motivational dimensions of setbacks. What remains striking is how consistently students prioritized interpersonal affirmation despite decades of research suggesting such messages might backfire or feel insincere. If we want professors to respond effectively when students struggle, we must equip them with language and practices that restore confidence while providing correction. Affirmation is not soft or indulgent. It is a form of informed encouragement that helps students recover from failure and reengage with learning.

## Figures and Tables

**Table 1:** Summary of key faculty survey results (effectiveness and commonness models).

Outcome	Significant Contrast/Predictor	Direction	β	SE	OR	95% CI	*p*
Effective	Validation vs. Sol Foc Resp	Validation > Sol Foc Resp	−1.56	0.43	0.21	[0.09, 0.46]	<0.001
	Affirmation vs. Sol Foc Resp	Affirmation > Sol Foc Resp	1.75	0.43	5.75	[2.48, 13.35]	<0.001
Frequent	Sol Focused Resp vs. Validation	Sol Foc Resp > Validation	0.95	0.42	2.58	[1.14, 5.84]	0.023
	Validation vs. Affirmation	Validation > Affirmation	−1.30	0.43	0.27	[0.12, 0.61]	0.003
	Sol Foc Resp vs. Affirmation	Sol Foc Resp > Affirmation	−2.25	0.43	0.11	[0.05, 0.24]	<0.001

Note. For brevity we used the term Sol Foc Resp to mean Solution-focused responses.

**Table 2:** Summary of key student survey results (effectiveness and commonness models).

Outcome	Significant Contrast/Predictor	Direction	β	SE	OR	95% CI	*p*
Effective	Affirmation vs. Validation	Affirmation > Validation	0.86	0.27	2.36	[1.36, 4.13]	0.001
	Affirmation vs. Sol Foc Resp	Affirmation > Sol Foc Resp	1.17	0.27	3.22	[1.91, 5.43]	<0.001
	Years Teaching → Validation	Fewer Years > More Years	−0.09	0.04	0.91	[0.84, 0.99]	0.022
Frequent	Sol Foc Resp vs. Validation	Sol Foc Resp > Validation	0.74	0.20	2.10	[1.39, 3.20]	<0.001
	Validation vs. Affirmation	Validation > Affirmation	−0.80	0.21	0.45	[0.29, 0.68]	<0.001
	Sol Foc Resp vs. Affirmation	Sol Foc Resp > Affirmation	−1.55	0.21	0.21	[0.14, 0.33]	<0.001

Note. For brevity we used the term Sol Foc Resp to mean solution-focused responses.

## Data Availability

The data for the study are available at [OSF link: https://osf.io/crpw4/overview?view_only=a2cf90bda1814042b90094c9dddcf090] (accessed on 5 December 2025).
